# Interplay of Substrate Retention and Export Signals in Endoplasmic Reticulum Quality Control

**DOI:** 10.1371/journal.pone.0015532

**Published:** 2010-11-24

**Authors:** Shinichi Kawaguchi, Chia-Ling Hsu, Davis T. W. Ng

**Affiliations:** 1 Temasek Life Sciences Laboratory, National University of Singapore, Singapore, Singapore; 2 Department of Biological Sciences, National University of Singapore, Singapore, Singapore; Universidade de São Paulo, Brazil

## Abstract

**Background:**

Endoplasmic reticulum (ER) quality control mechanisms are part of a comprehensive system to manage cell stress. The flux of molecules is monitored to retain folding intermediates and target misfolded molecules to ER-associated degradation (ERAD) pathways. The mechanisms of sorting remain unclear. While some proteins are retained statically, the classical model substrate CPY* is found in COPII transport vesicles, suggesting a retrieval mechanism for retention. However, its management can be even more dynamic. If ERAD is saturated under stress, excess CPY* traffics to the vacuole for degradation. These observations suggest that misfolded proteins might display different signals for their management.

**Methodology/Principal Findings:**

Here, we report the existence of a functional ER exit signal in the pro-domain of CPY*. Compromising its integrity causes ER retention through exclusion from COPII vesicles. The signal co-exists with other signals used for retention and degradation. Physiologically, the export signal is important for stress tolerance. Disabling it converts a benign protein into one that is intrinsically cytotoxic.

**Conclusions/Significance:**

These data reveal the remarkable interplay between opposing signals embedded within ERAD substrate molecules and the mechanisms that decipher them. Our findings demonstrate the diversity of mechanisms deployed for protein quality control and maintenance of protein homeostasis.

## Introduction

Protein biosynthetic pathways are normally at equilibrium with quality control mechanisms that monitor folding and assembly. The small fraction of maturation failures are segregated and delivered to degradative pathways like the ubiquitin-proteasome system (UPS). Under severe stress, when the proportion of unfolded proteins rises, the balance can shift to catabolism as a prophylactic strategy against toxicity. In the endoplasmic reticulum, these events are controlled by the unfolded protein response (UPR) (for reviews, see [1], [2]). In metazoans, the different outputs of the UPR allow for a staged response with the initial phase to favor restoration of folding capacity. If homeostasis is not restored, a catabolic stage ensues [3]. In the budding yeast *Saccharomyces cerevisiae*, the UPR is simpler and composed of the single, conserved Ire1 output. Here, it is an inducible pathway that regulates nearly 400 genes [4]. All aspects of protein folding and maturation as well as ER quality control are represented among these genes. Interestingly, these represent but a small fraction of the targets. The roles of many other functions regulated by the UPR remain unknown.

ER quality control mechanisms play a key role in ER stress tolerance. They monitor the folding states of newly synthesized proteins. Unfolded proteins are retained in the ER and those irreversibly misfolded are targeted to ER-associated degradation (ERAD) pathways (for review, see [5]–[7]). ERAD pathways are highly specialized mechanisms that incorporate the basic UPS at their core. As with most UPS substrates the final destruction signal is polyubiquitin, attached by specialized E3 ubiquitin ligase complexes embedded in the ER membrane. In budding yeast, the two known ERAD E3s, Hrd1p and Doa10p, modify distinct substrate classes. The Doa10p complex ubiquitinates membrane proteins with malformed cytosolic domains and soluble cytosolic proteins bearing specific destruction signals (8–11). The larger Hrd1p complex ubiquitinates proteins bearing transmembrane lesions and also damaged lumen-localized proteins/domains [8], [11]–[14]. All substrates are extracted from the ER by the associated Cdc48 complex before degradation by the 26S proteasome.

It is widely accepted that ERAD substrates are recognized and processed by ER receptor sites. However, it is also known that some molecules traffic to the Golgi before they are retrieved for ERAD [15]–[17]. Before degradation, N-linked oligosaccharides are released by the cytosolic peptide:*N*-glycanase (PNGase or Png1p) [18], [19]. These free oligosaccharides provide a biochemical record of endogenous substrates processed by ERAD-L (L, luminal). Suzuki and coworkers recently exploited this step of ERAD to show that a significant fraction was modified by the Golgi mannosyltransferase Och1p [20]. This analysis provides compelling evidence that substrate transport and retrieval is not restricted to models but a common mechanism of ERAD. Blocking these activities using yeast mutants also disrupts substrate degradation, suggesting a role in ERAD [15], [16]. However, it was proposed that the observed defects could be an indirect consequence of the particular mutant strains used [21]. Thus, whether the retrieval mechanism used by some substrates is also required for their ERAD remains unresolved.

Although some misfolded luminal proteins recycle between the ER and Golgi prior to ERAD, these can be diverted to the vacuole under conditions of severe stress [22]. The UPR regulates components of this pathway, which includes membrane trafficking mediators as well as vacuolar proteases [4]. Whether the ER-to-vacuole pathway is an essential facet of ER stress tolerance is not clear. Some evidence comes from strains lacking the cargo sorting receptor Erv29p. *ERV29* mutants cannot transport misfolded proteins and exhibit sensitivity to ER stress [15], [22]. On the other hand, they are also defective in the export of some normal proteins, which might indirectly compromise stress tolerance [23]. Thus, even as the trafficking of misfolded proteins through the endomembrane system is well documented, its physiological role is unclear and its underlying mechanisms relatively unexplored.

Two recent studies specifically explored the role of export signals in ER quality control. To determine the effect of a powerful ER export signal on the processing of a misfolded protein, Kincaid and Cooper engineered novel versions of CPY* fused to the transmembrane and cytosolic domains of Sys1p [24]. The Sys1p cytosolic domain contains a well-characterized diacidic motif export signal recognized by the Sec23/24 proteins of the COPII complex [25], [26]. Interestingly, the chimeric protein was efficiently transported from the ER, demonstrating that the strong Sys1p export signal could override the retention of CPY* by ER quality control. Transport was dependent on the export signal since its alteration caused the chimera to be retained. Adopting a different approach, Miller and coworkers studied the quality control of misfolded Yor1p (called Yor1p-ΔF), a homolog to the cystic fibrosis transmembrane conductance regulator [27]. Like the CPY*/Sys1p chimera, Yor1p sorting into COPII vesicles is dependent on a cytoplasmic diacidic motif. However, Yor1p-ΔF is retained in the ER, even when ERAD was blocked. Unresolved from these studies is whether embedded export signals are functional when the proteins are unfolded. A conformational requirement for the formation of ER export signals can underlie a workable retention mechanism for ER quality control. This mechanism may be in place for some proteins [28], [29]. Although appealing for its simplicity, it is certainly not a general mechanism for all molecules because there is clear evidence that some misfolded proteins traffic from the ER in COPII vesicles [22], [24], [30]–[34].

In this study, we examined the interplay between export signals and opposing retention/ERAD signals using the classical model substrate CPY*, a soluble luminal protein [35]. We determined that CPY could display both types of signals when misfolded. The export signal is not required for ERAD, which is sufficient to handle the protein load under low stress conditions. However, under severe stress, the export signal becomes an essential element to divert excess substrate to the UPR-regulated ER-to-vacuole degradative pathway. CPY* variants lacking their export signal are toxic due to their inability to utilize the alternative pathway.

## Results

### The model misfolded protein CPY* contains a functional ER exit signal

**Figure 1:**
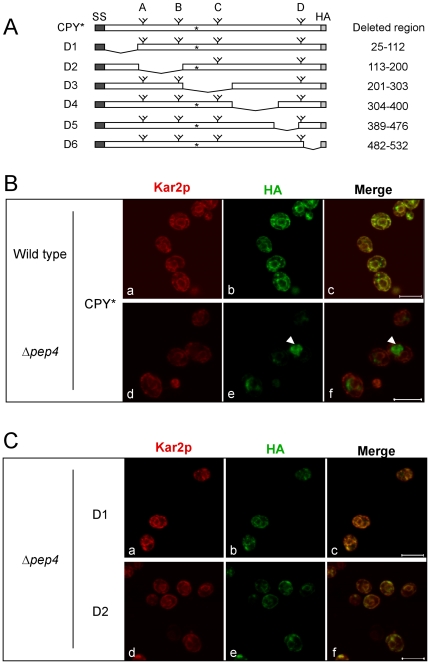
Analysis of CPY* export signals. (A) Schematic representation of CPY* and its deletion variants (D1–D6). Carbohydrate chains are shown by branched symbols, asterisks indicate the position of the G255R mutation, dark shaded boxes indicate signal sequences, and the HA epitope tag is shaded light gray. (B) Intracellular localization of highly expressed CPY* deletion variants in wild type and *Δpep4* strains. CPY* variants was detected using anti-HA antibody and visualized in the green channel. ER and nuclear envelope was visualized in the red channel using anti-Kar2p antiserum. (C) Intracellular localization of D1 and D2 variants in *Δpep4* cells. Substrates and ER/nuclei are visualized as in panel B. Localization of all CPY* deletion variants in both wild type and *Δpep4* cells are shown in Figure S1. Arrowhead indicates the accumulated CPY* or its variant in vacuole in a representative cell. Scale bars, 5 µm.

We sought to understand the mechanism and physiological significance of misfolded protein export from the ER. Previously, this phenomenon was studied using transport defective mutant strains [15], [16], [21]. However, indirect effects caused by impairment of normal cargo proteins could not be ruled out [21]. This drawback could be mitigated by modifying substrates to disable transport. To test the feasibility of the approach, CPY* deletion variants were created systematically to eliminate a potential export signal (Fig. 1A). It should be noted that no ER export signal is known for CPY* nor even wild type CPY. To facilitate analysis, all constructs contain an HA epitope-tag at their carboxy-termini, which does not affect ERAD nor transport [22]. In addition, the C-terminal glycan of CPY* (previously termed the “D-glycan”) is maintained in all variants because it is required for recognition by ERAD [36].

**Figure 2:**
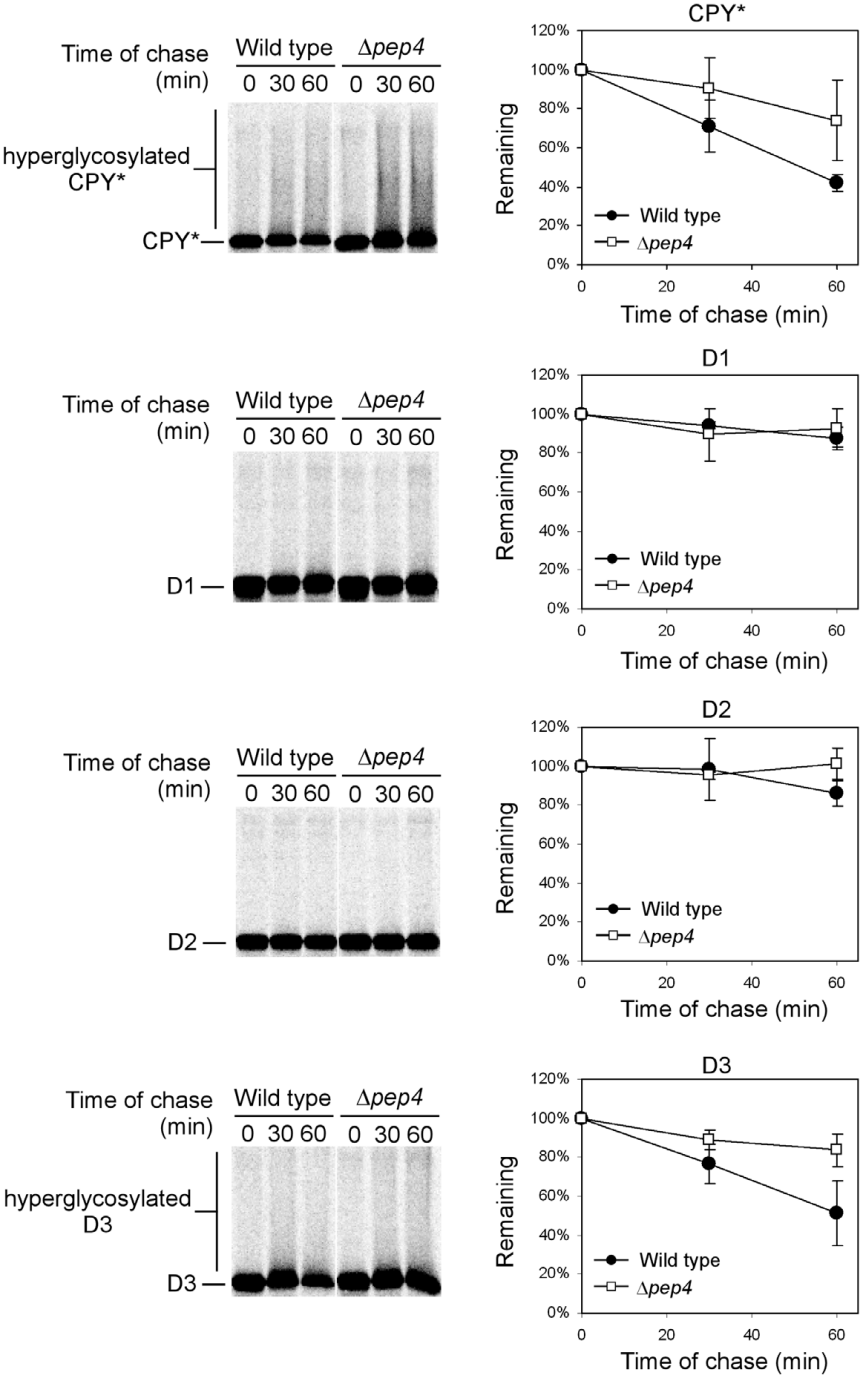
CPY* variants D1 and D2 are degradation defective. Wild type and *Δpep4* cells expressing CPY* and variants following galactose induction were pulse-labeled for 10 min with [^35^S]methionine/cysteine and chased for times indicated. Substrate proteins were immunoprecipitated from detergent lysates, separated by SDS-PAGE, and visualized and quantified by phosphorimager analysis. Representative gel scans are shown on the left. The position of substrate proteins and hyperglycosylated species are indicated. Data plots reflect three independent experiments with standard deviations indicated by the error bars.

We combined *in vitro* and *in vivo* approaches to analyze COPII vesicle packaging and vacuolar transport of CPY* variants, respectively. At moderate expression levels, CPY* is efficiently degraded by ERAD, with some molecules packaged into COPII vesicles and retrieved from the Golgi beforehand [16]. Expression of CPY* under the control of the strong *GAL1* promoter saturates ERAD and activates the UPR [22]. The UPR controls the ER-to-vacuole transport pathway, which is used to dispose excess CPY* under these conditions. Consistent with our previous observations, indirect immunofluorescence confocal imaging localizes CPY* to the ER of wild type cells (Figure 1B, upper panels). Vacuolar staining is absent because of rapid substrate degradation there. To visualize the fraction that traffics to the vacuole, substrates were also expressed in the vacuolar protease deficient *Δpep4* strain. Here, CPY* is detected strongly in compartments outside the ER that were previously determined to be vacuoles (Figure 1B, lower panels) [22]. All CPY* deletion variants display a similar pattern, except two (Figures 1C and S1). The CPY*-D1 and CPY*-D2 variants exhibited no detectable extra-endoplasmic reticulum staining indicating defects in ER export. In line with this view, the steady state levels of these substrates are significantly higher than CPY* and other variants in wild type cells (Figure S3A). Indeed, in pulse chase experiments, CPY*-D1 and CPY*-D2 constructs are stable proteins in wild type cells with no further stabilization in *Δpep4* cells (Figure 2). This result contrasts with transport competent CPY*, where a fraction is degraded by ERAD in *Δpep4* cells (Figure 2A) [22]. Because the portions deleted in CPY*-D1 and CPY*-D2 are not required for ERAD when moderately expressed [46], the data suggest that transport-defective CPY* variants can interfere with ERAD functions if highly expressed.

**Figure 3:**
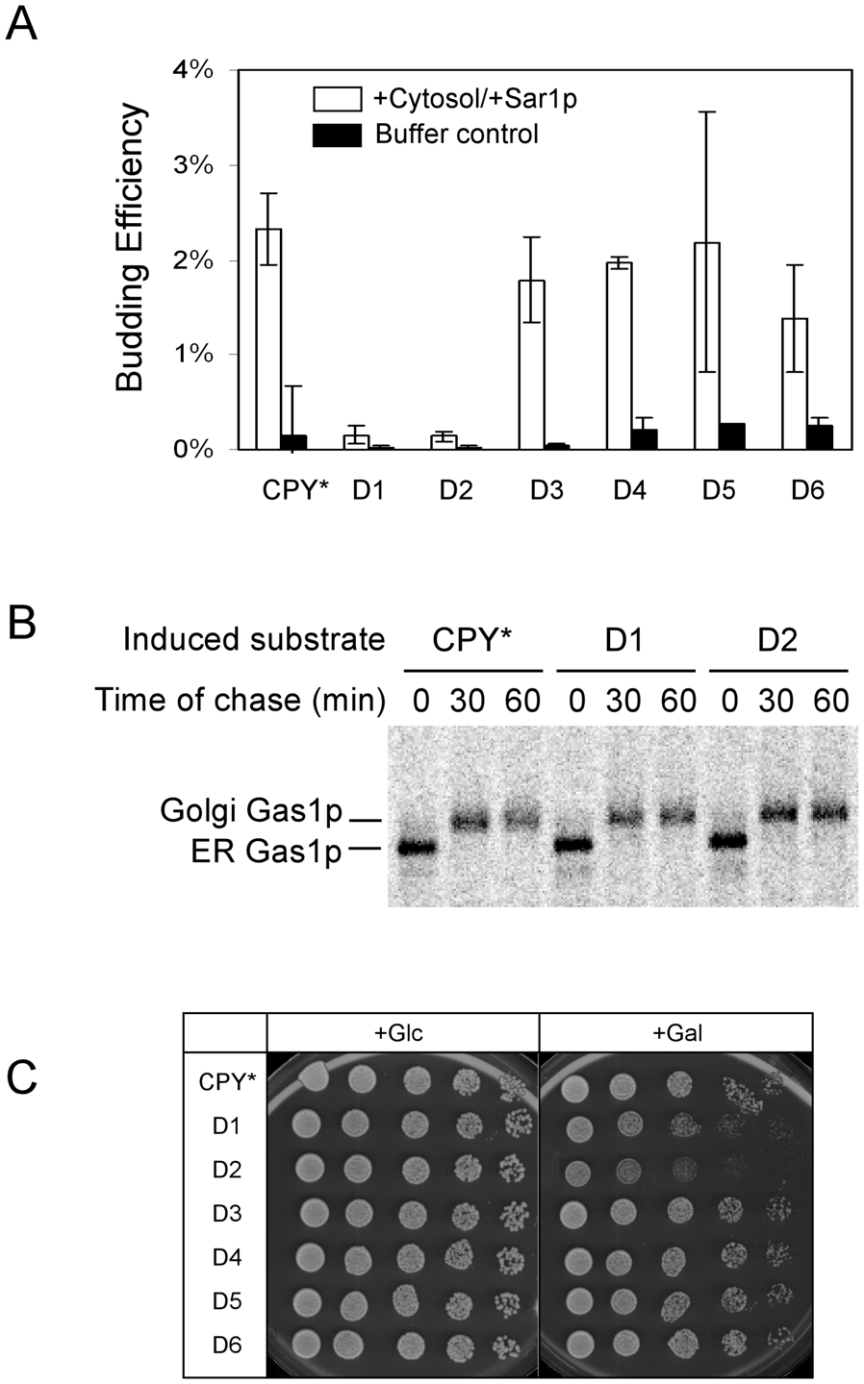
ER transport deficient CPY* variants are cytotoxic. (A) D1 and D2 variants are defective in ER vesicle budding. *In vitro* vesicle budding assays were performed using permeabilized cells from wild-type cells highly expressing CPY* and deletion variants. Total membranes and budded vesicles (Figure S2. “Sup”) were collected from each reaction containing cytosol/Sar1p or buffer only. Cargo packaging efficiency was analyzed by immunoblotting and quantified using the LI-COR fluorescence imaging system. A representative fluorograph is shown in Figure S2. Detection of the endogenous COPII vesicle protein, Erv25p, was included as a positive control. Three independent assays were performed for each experiment with error bars reflecting the standard deviation. Statistical significance was determined using Student's paired *t*-test (D1 or D2 vs. CPY* control, *p*<0.01). (B) Expression of D1 and D2 variants do not cause a general block in transport from the ER. Wild-type cells highly expressing CPY* and the D1 and D2 variants were pulse-labeled for 10 min with [^35^S]methionine/cysteine and chased for the indicated times. Endogenous Gas1p was immunoprecipitated from detergent lysate, separated by SDS-PAGE, and visualized by phosphorimaging. (C) Wild-type cells highly expressing CPY* or its variants were grown overnight in culture medium with 3% raffinose (pre-induction). Cells were spotted on SC plates containing 2% glucose (Glc, repressed) or 2% galactose (Gal, induced) as serial dilutions of each cell culture. Plates were incubated for 2 days at 30°C.

We next sought to determine how loss of the D1 and D2 segments disrupt CPY* trafficking. The simplest explanation posits that elements contained within them are required for ER export. Alternatively, the deletions might disrupt a vacuolar sorting signal resulting in secretion and/or retrieval of substrates. Pulse-chase analysis is consistent with the first scenario. Compared with CPY*, the D1 and D2 variants displayed little of the heterogeneous outer chain glycosylation characteristic of CPY* molecules trafficking through the Golgi (Figure 2, “hyperglycosylated” forms) [22]. To address the question directly, we applied an *in vitro* assay to test the packaging of CPY* variants into COPII vesicles. Semi-intact cells were prepared from wild-type strains expressing the appropriate CPY* variant. To initiate the reaction, cytosol from wild-type yeast cells and recombinant Sar1p were added along with GTP, GDP-mannose, and an ATP regeneration system [37]. Budded vesicles were recovered in the supernatant fraction, purified, and concentrated. Recovery of Erv25p (a constituent of COPII vesicles) in the vesicle fraction in the defined system and absent from control membranes demonstrates the efficacy of the assay (Average packaging efficiency, 8.9%. Figure S2A). Similarly, CPY* and the D3 through D6 variants can be packaged into COPII vesicles (Figures 3A and S2A). Likely due to high substrate levels, packaging efficiency of CPY*/variants was modest, yet nevertheless similar to previous studies using a purified microsome system [16]. By contrast, CPY*-D1 and CPY*-D2 variants were largely absent in the budded vesicle fraction (Figure 3A). These data provide an independent line of evidence that traces their transport defect to the ER vesicle budding step. To determine if CPY*-D1 and CPY*-D2 interferes with general vesicle trafficking, transport of the plasma membrane protein Gas1p was analyzed following their induction [38]. As shown in Figure 3B, Gas1p is transported from the ER efficiently in all situations after substrate induction indicating that the variants do not generally affect protein trafficking. These data show that the D1 and D2 lesions prevent the trafficking of excess CPY* to the vacuole through selective exclusion from COPII vesicles. The transport defects indicate that an ER export signal(s) was compromised by the D1 and D2 deletions.

Cell death caused by highly expressed CPY* in *IRE1* or *ERV29* mutants suggested that misfolded protein accumulation in the ER, if left unchecked, is toxic [22], [39]. However, it was unclear whether CPY* accumulation was the sole cause of death or if decreased fitness caused by null mutations also contributed. With export deficient CPY* variants, it is now possible to answer this question directly without using genetically compromised cells. For this, we induced CPY* and variant expression in wild type cells. As shown in Figure 3C (+Glc), all strains grew well on glucose media which represses expression of the misfolded proteins. A different pattern emerged following induction on galactose media. Cells highly expressing CPY* grew well as previously reported [22]. Variants D3 through D6 exhibited no inhibition compared with full-length CPY*. By contrast, the growth of cells expressing CPY*-D1 and CPY*-D2 was strongly inhibited (Figure 3C, rows D1 and D2). Strikingly, *Δcue1* and *Δhrd1* cells (required for luminal ERAD [40]–[42]) grew well when expressing CPY* and transport competent variants (Figure S4, +Gal panel). This result shows that availability of the ER-to-vacuole degradative pathway alone can alleviate their toxicity. As expected, expression of transport-deficient variants was toxic to these cells. These data show that the potential toxicity of misfolded CPY is mitigated by a functional ER export signal in the unfolded molecule. Without the ability to exit the ER, misfolded variants become toxic even in wild type cells. This finding provides a physiological basis for the existence of functional ER export signals in misfolded proteins.

### N-linked glycans are required for CPY* trafficking

We wondered what features of the deleted sequences are required for export. The D1 lesion removed the pro-domain of CPY but left the mature portion intact. This finding was particularly exciting because another Erv29p-dependent cargo protein, folded glycopro-α factor, contains its ER export signal in the pro-domain [43]. Although the pro-domain is required, it may not be sufficient because the D2 deletion also blocks transport. Unlike the deleted D1 sequences, the deleted segment of CPY*-D2 is normally modified by two N-linked glyans in full length CPY* (Figure 1A). Because N-linked glycosylation is required for the transport of some proteins [44], we analyzed the contribution of three glycans not involved in ERAD signaling (Figure 1A: glycans A, B, and C) systematically.

**Figure 4:**
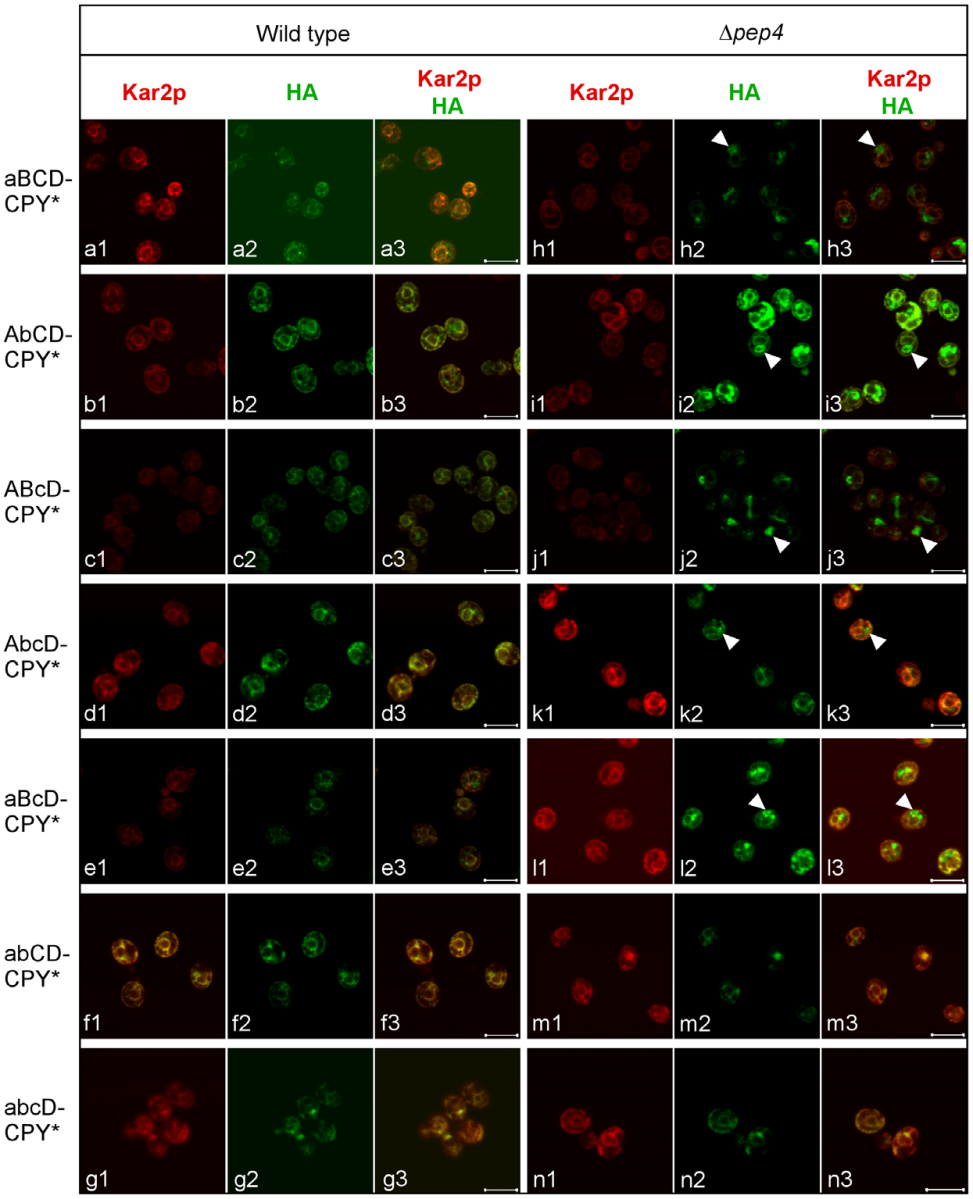
N-glycans are required for for CPY* vacuolar transport. Wild type and *Δpep4* cells carrying CPY* and its glycosylation variant genes (wild type glycan sites are denoted in upper case, mutant sites in lower case) were induced for 6 hr. Intracellular localization of proteins were performed by indirect immunofluorescence as in Figure 1. Arrowheads indicate vacuolar forms of induced proteins. Scale bars, 5 µm.

By site-directed mutagenesis, the three glycosylation sites were eliminated singly (lower case letters indicate mutated sites: aBCD-CPY*, AbCD-CPY*, and ABcD-CPY*), doubly (abCD-CPY*, aBcD-CPY*, and AbcD-CPY*), or together (abcD-CPY*, previously constructed (36)). Expression was induced with galactose and each variant was localized by indirect immunofluorescence in wild type and *Δpep4* cells (Figure 4). Like CPY*, single glycan mutants were found in vacuolar compartments indicating that no single glycan is essential for export (Figure 4, upper 3 rows). The AbCD-CPY* variant showed greater ER staining than the others suggesting that the B glyan might contribute most significantly to ER exit (Figure 4, compare panel i3 to h3 and j3). Among double mutants, the strong vacuolar localization of the aBcD-CPY* confirms the importance of the B-glycan (Figure 4, compare panel l3 to k3 and m3). AbcD-CPY* localized to vacuolar compartments but much more weakly than aBcD-CPY* (Figure 4, compare panels l3 and k3). With abCD-CPY*, however, no vacuolar localization could be detected. These data indicate that the A and B glycans, eliminated in the D2 variant, are essential for vacuolar transport. Accordingly, the abcD-CPY* variant was localized exclusively to the ER (Figure 4, panels n1 to n3).

**Figure 5:**
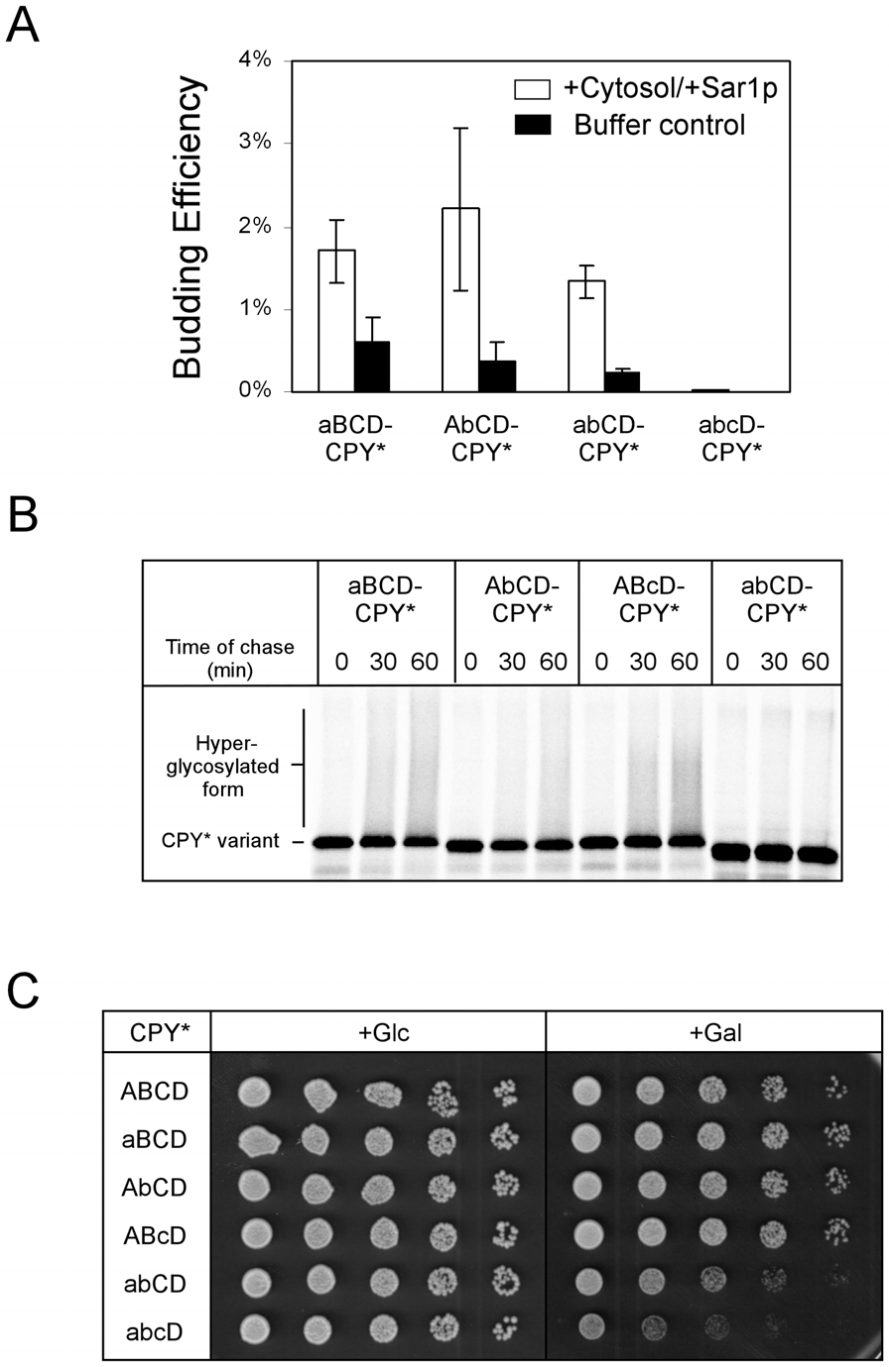
Glycans A and B are required for ER export. (A) Wild-type cells highly expressing aBCD-CPY*, AbCD-CPY*, abCD-CPY*, and abcD-CPY* were used in *in vitro* vesicle budding assays as described in Figure 3. Each data set is from three independent experiments with the standard deviation represented by error bars. Statistical significance was determined using Student's paired *t*-test (aBCD-CPY*, AbCD-CPY*, or abCD-CPY* vs. abcD-CPY*, *p*<0.05). (B) *Δpep4* cells highly expressing glycan variants in panel A were pulse-labeled for 10 min with [^35^S]methionine/cysteine and chased as indicated. Substrate proteins were immunoprecipitated, separated by SDS-PAGE, and visualized by phosphorimaging. The position of hyperglycosylated forms is indicated. (C) Transport deficient glycan variants are toxic. Wild type cells carrying CPY* (ABCD) and glycan variant genes were assayed for cytotoxicity following induction as described in Figure 3C.

To analyze further the role of the A, B, and C glycans in CPY* transport, COPII vesicle budding assays were performed on aBCD-CPY*, AbCD-CPY*, abCD-CPY*, and abcD-CPY* (Figure 5A and S2B). Single glycosylation mutants packaged into COPII vesicles to an extent similar to fully glycosylated CPY* (compare Figure 5A with Figure 3A). The abCD-CPY* variant was packaged less efficiently and the variant lacking all three sites was undetected in the vesicle fraction (Figure 5A). Although the trend is in good agreement with localization studies, the requirements for CPY* vesicle budding *in vitro* are less stringent for the presence of individual glycans. To reconcile these differences, we analyzed the effects of glycan site mutations by pulse-chase analysis in *Δpep4* cells, which do not impede transport but do prevent vacuolar degradation. We adapted this *in vivo* assay to measure transport by monitoring conversion to the heterogeneous migrating forms, a measure of transport through the Golgi. Using this approach, abCD-CPY* converted forms were not detected indicating a block in ER-to-Golgi transport (Figure 5B). For the single mutants, aBCD-CPY* and ABcD-CPY* mutants were converted relatively efficiently with AbCD-CPY* falling somewhere in between. Taken together, these data show that N-linked glycans are required for CPY* ER export with glycan B being the most important.

To determine further whether transport efficiency correlated with toxicity, CPY* glycosylation mutants were expressed in wild type cells by galactose induction. Here, cells challenged with single-glycan mutants grew no worse than control (Figure 5C). By contrast, the transport defective abCD-CY* and abcD-CPY* variants caused strong growth inhibition. The slightly stronger inhibition caused by abcD-CPY* supports the idea that the C glycan can also contribute to transport. Importantly, Western analysis shows much greater steady state levels of these variants in wild type cells compared with transport competent variants (Figure S3B). Unlike CPY* deletion variants, these have equal lengths and differed primarily by glycan occupancy. These data strengthen the conclusion that the ability of substrates to use the ER-to-vacuole pathway reduces their intrinsic toxicity.

### Transport and retrieval are not prerequisites for ERAD

**Figure 6:**
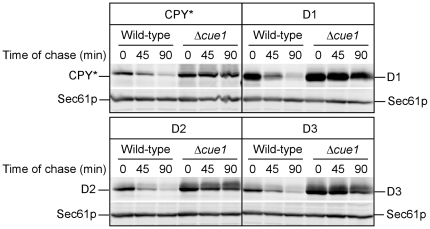
Export deficient mutants are efficiently degraded by ERAD. CPY* and the D1, D2, and D3 variants were moderately expressed under the control of its native promoter in wild type or *Δcue1* cells. Equal cell numbers were harvested at the indicated times after the addition of cycloheximide (100 µg/ml) and detergent lysates prepared. Proteins were separated by SDS-PAGE and detected by immunoblotting. Quantification was performed using an Odyssey infrared imaging system (LI-COR Biosciences, Lincoln, NE). Endogenous Sec61p was detected from the same filters as a loading control.

CPY*, expressed at moderate levels from its native promoter, is degraded exclusively by ERAD [45]. Under these conditions, some molecules are packaged into COPII vesicles indicating that they are degraded after their retrieval from the Golgi apparatus [16]. The stabilization of CPY* in COPII and COPI vesicle transport mutants suggested that trafficking might be a requirement for its degradation [15], [16]. However, it was proposed that the strong stabilization observed might be due to secondary effects of the transport mutants on ERAD [21]. The CPY*-D1 and CPY*-D2 variants can be used to resolve this issue because they carry the CPY ERAD determinant and are unable to exit via the COPII pathway [46]. To determine if transport and retrieval is coincidental or a requirement for ERAD, the D1, D2, and D3 variants were expressed moderately from the *PRC1* (CPY) promoter and turnover was analyzed by cycloheximide chase and immunoblotting. The CPY* control is degraded rapidly in wild type cells and stabilized in the *Δcue1* ERAD mutant as expected (Figure 6, upper left). The transport competent CPY*-D3 variant behaves identically showing that a large internal deletion has no effect on degradation as long as its ERAD determinant is present (Figure 6, lower right). Applying the same assay to CPY*-D1 and CPY*-D2, their turnover profile is identical to CPY*. These data show that substrate transport and retrieval are not requirements for ERAD. Instead, the observed transport and retrieval of misfolded proteins likely reflects a mechanism of ER retention, analogous to that of ER resident proteins bearing C-terminal HDEL retention sequences [47].

## Discussion

Proteins exported out of the ER are concentrated into COPII-coated vesicles through interactions with cargo sorting receptors. Export signals include diacidic or dihydrophobic motifs located in the cytoplasmic domains of some membrane proteins. These are recognized by the Sec23/24 subunits of the COPII complex [25], [26]. Physically separated from coat components, soluble cargo proteins are sorted by binding various export receptors in the ER lumen [48]. Although not as well characterized, soluble cargo proteins including procathepsin Z and alkaline phosphatase contain export signals that are functional only when folded [28], [29]. These findings led to proposals that the formation of ER export signal dependent on polypeptide folding could be a general mechanism of ER quality control. However, evidence that some molecules can efficiently traffic in the absence of positive acting export signals has challenged this view [49].

In this study, we demonstrated that the classical ERAD substrate CPY* contains determinants for ER export that encompass the pro domain and at least one nearby glycan. Although our data do not rule out a local conformational component to the signal, its ability to function does not depend on the correct overall structure of the protein. Importantly, the export signal does not subvert ERAD. Instead, the presence of chaperone binding sites and positive ERAD signals are sufficient to retain and degrade CPY* [46]. In this way, CPY* differs from the transmembrane protein Wsc1p, which contains a powerful ER export signal in its cytoplasmic domain. When its luminal domain misfolds, the lack of an ERAD degradation/retention signal makes it dependent on a post-ER mechanism for quality control [30]. During times of stress, however, CPY*'s ER export signal allows it to bypass ERAD and use the ER-to-vacuole pathway for turnover [22].

The A, B, and C glycans contribute to CPY* ER export, with the B glycan being the most important. Currently, their role is unclear. By analogy to glycan-dependent ERAD, the signal could be bipartite, combining pro-domain sequences and a glycan for recognition by an unknown lectin-like cargo receptor [46]. Alternatively, because the glycans can substitute for each other, they may instead play a structural role that promotes CPY* solubility. Along a similar line, these glycans could contribute to the folding of a conformational export signal. In this scenario, the glycans are not part of a signal but contribute to its formation. Glycans are not just important for the export of misfolded CPY. A detailed study of CPY N-linked carbohydrates revealed that the B-glycan is critical for transport of the folded molecule. An AbCD-CPY variant is transport deficient while a variant carrying just the B glycan is transported efficiently [50]. The D glycan on the other hand, required for ERAD of misfolded CPY, is entirely dispensable for transport and enzymatic activity. These studies demonstrate the diversity of functions of individual N-linked glycans of CPY for biogenesis, transport, and quality control.

High-level expression of CPY* is highly toxic to transport-deficient strains but it does not affect the apparent fitness of wild-type cells [22], [39]. CPY* accumulation in *Δerv29* cells resulted in the generation of reactive oxygen species that likely contributes to cell death [39]. Erv29p is a COPII vesicle cargo sorting receptor that is required to package a number of endogenous folded proteins including proCPY and pro-α factor [23]. To what extent the general trafficking block contributed to the observed phenotype is unknown. In this study, disabling the CPY* ER export signal converted a well-tolerated protein to one that is toxic. This difference pinpoints the nature of the proteotoxcity and leaves no doubt that the ER accumulation of an ERAD substrate, beyond a certain threshold, is highly toxic. Our data show that the functional export signal in misfolded CPY contributes to ER stress tolerance by allowing it to use the vacuolar pathway. Whether functional export signals are generally found in misfolded proteins is not known.

The ER-to-vacuole pathway is well suited for a role in stress tolerance because it has a greater substrate capacity than ERAD [22]. Recent reports have revealed the vacuole/lysosome plays a major constitutive role in the disposal of aberrant proteins. Protein aggregates in the cytosol and ER are delivered to the vacuole/lysosome using autophagic pathways [51]–[53]. Some misfolded proteins that evade ER quality control are transported the vacuole using the classical secretory pathway [22], [30]–[34], [54]. The studies show that the vacuole/lysosome system is at least an equal partner to the UPS in the disposal of aberrant proteins.

Export-deficient CPY* variants were degraded by ERAD if moderately expressed. This shows that trafficking between the compartments is not a prerequisite for ERAD. Why then, do some CPY* molecules traffic to the Golgi before ERAD? Our data show that CPY* can display signals for both COPII-mediated export and ERAD. Efficient degradation of transport-defective CPY* shows that ER export and ERAD are not obligatorily sequential steps, but kinetically competitive processes. Thus, substrate retrieval from the Golgi might simply reflect an ER retention mechanism similar to proteins carrying C-terminal HDEL signals [47]. Indeed, the CPY* ERAD determinant is a binding site for Kar2p/BiP [46], an HDEL-bearing ER chaperone [55], [56]. Thus, the presence of functional ER export signals makes a retrieval mechanism essential for the ERAD of molecules bearing them.

Recent findings from the Suzuki laboratory suggest that the trafficking of misfolded proteins to the Golgi might play a more active role in ERAD. An analysis of free cytosolic oligosaccharides (released from endogenous ERAD substrates by PNGase) showed a low abundance of GlcNAc_2_Man_7_ glycans [20]. This is significant because the glycoform is generated by Htm1p, a mannosidase whose activity exposes a terminal α1,6-linked mannose that serves as a ligand for the ERAD factor Yos9p [57], [58]. Surprisingly, a much larger proportion of free oligosaccharides also bears the potential Yos9p ligand but generated instead by the Golgi localized Och1p mannosyltransferase. This raises the exciting possibility that Och1p functions analogously to Htm1p. In this mode, misfolded glycoproteins that traffic to the Golgi would receive terminal α1,6-linked mannose signal before their return to the ER for ERAD.

In summary, our studies using the classical ERAD model substrate CPY* show that functional ER export signals coexist with ERAD/retention signals. Remarkably, these signals are interpreted by the quality control machinery to use the ERAD pathway under normal conditions. Under ER stress, concomitant with increasing misfolded protein concentrations and UPR activation, these signals are used to detoxification by diverting the molecules to the vacuole.

## Materials and Methods

### Plasmids

Plasmids used in this study are listed in Table 1 and were constructed using standard cloning protocols [59]. All plasmid inserts were confirmed by DNA sequence analysis. pDN436 encodes CPY*-HA under the control of *PRC1* promoter [60]. A CPY*-HA deletion series was constructed as described below. D1, D2, D3, D4, D5, and D6 variants lack amino acid residues 25–112, 113–200, 201–303, 304–400, 389–476, and 482–532 of CPY*-HA, respectively.

**Table 1:** Plasmids used in this study.

Plasmid	Protein	Carbohydrate Modification	Promoter	Vector	Source
pES28	CPY*	ABCD	*GAL1*	pTS210	Spear and Ng (2003)
pSK88	D1	ABCD	*GAL1*	pTS210	This study
pSK89	D2	CD	*GAL1*	pTS210	This study
pSK90	D3	ABD	*GAL1*	pTS210	This study
pSK91	D4	ABCD	*GAL1*	pTS210	This study
pSK92	D5	ABCD	*GAL1*	pTS210	This study
pSK93	D6	ABCD	*GAL1*	pTS210	This study
pSK95	CPY*	aBCD	*GAL1*	pTS210	This study
pSK96	CPY*	AbCD	*GAL1*	pTS210	This study
pSK97	CPY*	ABcD	*GAL1*	pTS210	This study
pSK94	CPY*	abCD	*GAL1*	pTS210	This study
pSK103	CPY*	AbcD	*GAL1*	pTS210	This study
pSK104	CPY*	aBcD	*GAL1*	pTS210	This study
pCH66	CPY*	abcD	*GAL1*	pTS210	Ling
pDN436	CPY*	ABCD	*PRC1*	pRS315	Ng *et al*. (2000)
pAS67	D1	ABCD	*PRC1*	pRS316	This study
pAS63	D2	CD	*PRC1*	pRS315	This study
pAS64	D3	ABD	*PRC1*	pRS315	This study
pAS77	D4	ABCD	*PRC1*	pRS315	This study
pAS68	D6	ABCD	*PRC1*	pRS316	This study

#### pAS63, pAS64

For each plasmid, a fragment containing the N-terminal coding sequences of CPY* behind the *PRC1* promoter was amplified by PCR using Vent DNA Polymerase (New England Biolabs, Ipswich, MA) with pDN436 as a template and cleaved with *Eag*I. The sequences encoding the C-terminal region of CPY* was similarly amplified and cleaved with *Xba*I. The phosphorylated fragments were ligated into the *Eag*I/*Xba*I sites of pRS315 to generate each plasmid. PCR primers were designed to precisely delete defined sequences after ligation into plasmids.

#### pAS67

Constructed as described in the preceding section except amplified products encoding CPY*-HA C-terminal sequences were digested with *Sal*I. The fragments encoding the N- and C-termini were ligated into the *Eag*I/*Sal*I sites of pRS316 to complete the deletion construct.

#### pES28

HA epitope-tagged CPY* behind the control of the *GAL1* promoter and inserted in YCp50 was previously described [22].

#### pSK88, pSK89, pSK90, pSK91, pSK93

CPY*-HA deletion variant coding sequences were amplified by PCR using pAS67, pAS63, pAS64, pAS77, or pAS68 as templates. Fragments were ligated directly into the T/A-cloning site of pYES2.1 vector containing *GAL1* promoter. These constructs were cleaved with *Age*I and *Sph*I. DNA fragment containing *GAL1* promoter and CPY* deletion was ligated into *Age*I/*Sph*I sites of the YCp50-based pTS210 vector [61].

#### pSK92

The *GAL1* promoter regulated CPY* variant D5 was created by deleting the coding region for 389–476 residues of CPY* via site-directed mutagenesis using pES28 as a template [62].

#### pSK95, pSK96, pSK97

The plasmids encoding the single glycosylation mutants aBCD-CPY*, AbCD-CPY*, and ABcD-CPY* in pRS315 were described previously [36]. The coding sequences of each variant was amplified by PCR and ligated directly into T/A-cloning site of pYES2.1 vector. The genes now behind the *GAL1* promoter were cleaved with *Age*I and *Sph*I and ligated into same sites in pTS210.

#### pCH66

The *GAL1* promoter regulated HA-tagged abcDCPY* vector, was constructed by digesting pES147 [36] with *Acc*I, followed by treatment with T4 DNA polymerase and digestion with *Sph*I. The released insert was ligated into *Bam*HI (T4 DNAP-treated)/*Sph*I-digested pTS210 [61].

#### pSK94, pSK103, pSK104

The plasmids for abCD-CPY*, AbcD-CPY*, and aBcD-CPY* were created via PCR-based mutagenesis using the pCH66 as a template by mutating codons encoding Asn to Gln in the corresponding N-glycosylation sites.

### Strains and antibodies

*Saccharomyces cerevisiae* strains used in this study are described in Table 2. Anti-HA monoclonal antibody (HA.11) was purchased from Covance Research Products (Richmond, CA). Anti-Kar2p and anti-Sec61p rabbit antisera were provided by Dr. Peter Walter (University of California, San Francisco, CA). Anti-Gas1p rabbit antiserum was previously described [22]. Anti-Erv25p antibody was a gift from Dr. Charlie Barlowe (Dartmouth College Medical School, Hanover, NH).

**Table 2:** Strains used in this study.

Strain	Genotype	Source
W303a	*MAT*a, *leu2-3,112*, *his3-11*, *trp1-1*, *ura3-1*, *can1-100*, *ade2-1*	P.Walter (UCSF)
ESY258	*MAT*a, pDN436, W303 background	Spear and Ng, 2003
ESY259	*MAT*a, *cue1*::*TRP1*, pDN436, W303 background	Spear and Ng, 2003
ASY208	*MAT*a, pAS67, W303 background	This study
ASY209	*MAT*a, *cue1*::*TRP1*, pAS67, W303 background	This study
ASY202	*MAT*a, pAS63, W303 background	This study
ASY203	*MAT*a, *cue1*::*TRP1*, pAS63, W303 background	This study
ASY200	*MAT*a, pAS64, W303 background	This study
ASY201	*MAT*a, *cue1*::*TRP1*, pAS64, W303 background	This study
SKY204	*MAT*a, pES28, W303 background	This study
SKY226	*MAT*a, pSK88, W303 background	This study
SKY227	*MAT*a, pSK89, W303 background	This study
SKY228	*MAT*a, pSK90, W303 background	This study
SKY229	*MAT*a, pSK91, W303 background	This study
SKY230	*MAT*a, pSK92, W303 background	This study
SKY231	*MAT*a, pSK93, W303 background	This study
SKY249	*MAT*a, pSK95, W303 background	This study
SKY250	*MAT*a, pSK96, W303 background	This study
SKY251	*MAT*a, pSK97, W303 background	This study
SKY255	*MAT*a, pSK94, W303 background	This study
SKY265	*MAT*a, pSK103, W303 background	This study
SKY267	*MAT*a, pSK104, W303 background	This study
CHY535	*MAT*a, pCH66, W303 background	This study
SKY232	*MAT*a, *pep4*::*HIS3*, pES28, W303 background	This study
SKY233	*MAT*a, *pep4*::*HIS3*, pCH66, W303 background	This study
SKY234	*MAT*a, *pep4*::*HIS3*, pSK88, W303 background	This study
SKY235	*MAT*a, *pep4*::*HIS3*, pSK89, W303 background	This study
SKY236	*MAT*a, *pep4*::*HIS3*, pSK90, W303 background	This study
SKY237	*MAT*a, *pep4*::*HIS3*, pSK91, W303 background	This study
SKY238	*MAT*a, *pep4*::*HIS3*, pSK92, W303 background	This study
SKY239	*MAT*a, *pep4*::*HIS3*, pSK93, W303 background	This study
SKY252	*MAT*a, *pep4*::*HIS3*, pSK95, W303 background	This study
SKY253	*MAT*a, *pep4*::*HIS3*, pSK96, W303 background	This study
SKY254	*MAT*a, *pep4*::*HIS3*, pSK97, W303 background	This study
SKY256	*MAT*a, *pep4*::*HIS3*, pSK94, W303 background	This study
SKY266	*MAT*a, *pep4*::*HIS3*, pSK103, W303 background	This study
SKY268	*MAT*a, *pep4*::*HIS3*, pSK104, W303 background	This study
SKY240	*MAT*a, *hrd1*::*KANMX*, pES28, W303 background	This study
SKY242	*MAT*a, *hrd1*::*KANMX*, pSK88, W303 background	This study
SKY243	*MAT*a, *hrd1*::*KANMX*, pSK89, W303 background	This study
SKY244	*MAT*a, *hrd1*::*KANMX*, pSK90, W303 background	This study
SKY245	*MAT*a, *hrd1*::*KANMX*, pSK91, W303 background	This study
SKY246	*MAT*a, *hrd1*::*KANMX*, pSK92, W303 background	This study
SKY247	*MAT*a, *hrd1*::*KANMX*, pSK93, W303 background	This study
SKY398	*MAT*a, *cue1*::*TRP1*, pES28, W303 background	This study
SKY399	*MAT*a, *cue1*::*TRP1*, pSK88, W303 background	This study
SKY400	*MAT*a, *cue1*::*TRP1*, pSK89, W303 background	This study
SKY401	*MAT*a, *cue1*::*TRP1*, pSK90, W303 background	This study
SKY402	*MAT*a, *cue1*::*TRP1*, pSK91, W303 background	This study
SKY403	*MAT*a, *cue1*::*TRP1*, pSK92, W303 background	This study
SKY404	*MAT*a, *cue1*::*TRP1*, pSK93, W303 background	This study

### Substrate induction and metabolic pulse-chase

Cells carrying *GAL1* promoter regulated CPY* and its variants were grown at 30°C in synthetic complete (SC+3% raffinose) media lacking the appropriate amino acids to mid-logarithmic phase. To initiate expression, cells were pelleted by low speed centrifugation, the supernatant discarded, and resuspended in fresh media containing 2% galactose. Cells were then grown for 6 h at 30°C before processing. Cell labeling, preparation of detergent lysates, and immunoprecipitation were carried out as described previously [16].

### Cycloheximide chase assay

Cells are grown as described in figure legends. To begin the chase, protein synthesis is terminated by adding cycloheximide to the culture media (100 µg/ml final) with continued incubation at 30°C. At specified time points, cells were collected and a tenth volume of ice-cold 100% trichloroacetic acid (TCA) added to end the chase. Cells were disrupted using 0.5 mm zirconium beads in a Mini Beadbeater-8 homogenizer at 2×30 s cycles (BioSpec Products Inc., Bartlesville, OK). TCA precipitates were collected by centrifugation and detergent lysates were prepared as described previously [16]. Proteins were separated by SDS-PAGE and transferred to nitrocellulose membranes. Blocked membranes were incubated with appropriate primary antibodies for 1 h at room temperature. Membranes were washed in phosphate buffered saline containing 0.1% Tween 20 and further incubated with secondary antibodies labeled with infrared dye, IRDye 680 or IRDye 800. Fluorescence was detected and quantified by using the Odyssey infrared imaging system (LI-COR Biosciences, Lincoln, NE).

### COPII vesicle budding assay using semi-intact cells

Permeabilized cell-based vesicle budding assays were performed as described previously [37] with some modifications. Galactose-activated CPY* and variants were spheroplasted and gently frozen at −80°C. Next, the frozen spheroplasts were gently thawed on ice and treated with 2.5 M Urea in B88 (20 mM HEPES/KOH, pH 6.8, 150 mM KOAc, 5 mM MgOAc, and 250 mM sorbitol) on ice for 5 min and washed with B88. Budding reactions (total 150 µl) contained 30 µl of the permeabilized cell membranes (from ∼3 OD_600_ cells), 300 µg crude cytosol, 3 µg the recombinant Sar1p, ATP mix (1 mM ATP, 40 mM creatine phosphate, and 0.2 mg/ml creatine phosphokinease in B88), 0.2 mM GTP, and 25 µM GDP-mannose. A fraction of each reaction mixture was saved as a control (total fraction). Reaction mixes were incubated at 30°C for 90 min and centrifuged at 14,000 rpm at 4°C for 2 min to separate budded vesicles. The vesicle fraction was further purified by a density step gradient as described previously [63] with following modifications. 125 µl of supernatant was transferred to a TLS-55 ultracentrifuge tube (Beckman Coulter, Brea, CA) and mixed with the same volume of B88. 250 µl of 70% w/v Nycodenz (Sigma-Aldrich, St. Louis, MO) in B88 was added and mixed. 500 µl each of 30%, 25%, and 15% Nycodenz in B88 were layered. The gradient was centrifuged at 50,000 rpm for 2 hr at 4°C. 100 µl from the top was discarded and the next 800 µl was placed in a new TLS-55 tube. Sample was diluted by adding equal volume of B88 and centrifuged again at 34,000 rpm for 1 hr at 4°C. After removal of all supernatant, membrane pellet was dissolved 31 µl of sample loading buffer for SDS-PAGE analysis (budded vesicle fraction). Protein detection and quantification were performed as described above by using the Odyssey infrared imaging system (LI-COR Biosciences, Lincoln, NE).

### Indirect immunofluorscence microscopy

Indirect immunofluorescence was performed as described previously [22]. Briefly, cells were fixed with 3.7% formaldehyde at 30°C for 90 min and spheroplasted by treating with 1 mg/ml zymolyase 20 T (United States Biological Inc., Marblehead, MA) in a 50 mM potassium phosphate buffer, pH 7.5 containing 1.4 M sorbitol for 20 min at room temperature. A cell suspension was applied to each well of a poly-L-lysine-coated slide for 10 min and the unbound cells were washed out. Slides were immersed in methanol for 6 min and in acetone for 30 sec at −20°C. Each well was blocked with TBS (50 mM Tris-Cl, pH 7.4, 150 mM NaCl) containing 0.05% Tween-20 and 5% non-fat dry milk. Primary antibodies and secondary antibodies were applied with appropriate dilution and incubated for 90 min each time. Slides were washed twice with TBS buffer after each application. Working concentrations of primary antibodies HA.11 mAb (Covance Research Products, Princeton, NJ) and polyclonal anti-Kar2p were 1∶200 and 1∶500 respectively. Secondary antiboldies Alexa Fluor 488 goat anti-mouse and Alexa Fluor 596 goat anti-rabbit (Molecular Probes, Inc., Eugene, OR) were diluted to 1∶500. Confocal images were obtained with Zeiss Axiovert 200 M microscope equipped with 100x 1.4NA oil Plan-Aprochromat objective (Carl Zeiss MicroImaging, Inc., Oberkochen, Germany). Images were archived by LSM Image Browser.

### CPY* proteotoxicity assay

Cells containing *GAL1* promoter regulated CPY* and its variants were grown at 30°C in synthetic media containing the appropriate amino acids and 3% raffinose to mid logarithmic phase. Cell densities were adjusted to 0.1 OD_600_ units/ml. The culture was diluted at 1∶10 serially 4 times and 5 µl was taken from each and spotted on SC plates containing 2% glucose (Glc) or 2% galactose (Gal). Plates were incubated for 2 days at 30°C.

## Supporting Information

Figure S1 Localization of CPY* deletion variants in wild type and ***Δpep4*** **strains.** Indirect immunofluorescence labeling as described in Figure 1, panels B and C. This figure shows the data set for wild type and all deletion constructs. The images shown in Figure 1 are included here to simplify viewing. Arrowheads show positions of substrate proteins localized in vacuoles.
(TIF)

Figure S2 ***In vitro*** vesicle budding assays of CPY* deletion and glycan mutants. (A) Representative immunoblots of data presented in Figure 3A. (B) Representative immunoblots of data presented in Figure 5A. The COPII vesicle membrane protein Erv25p is detected as a positive control for vesicle budding in each experiment (23).
(TIF)

Figure S3 Relative expression levels of CPY* variants in wild-type cells. (A) CPY* and deletion variants were expressed in wild type cells for 6 h following galactose induction. Protein extracts from each strain were separated by SDS-PAGE and transferred onto a nitrocellulose membrane. Substrate proteins were detected using anti-HA antibody. Visualization was performed using fluorescent secondary antibodies as described in *Materials and Methods*. The detection of Sec61p on the same membrane was used as a loading control. The positions of molecular weight markers are indicated. (B) CPY* and glycan variants were analyzed as described in panel A.
(TIF)

Figure S4 **Substrate toxicity in ERAD mutants.** *Δcue1* and *Δhrd1* cells carrying CPY* and deletion variant genes were grown overnight in culture medium containing 3% raffinose. Each culture was spotted onto agar plates as serial dilutions as described in Figure 3C.
(TIF)
