# Supplementary figures and images for: Interplay of Substrate Retention and Export Signals in Endoplasmic Reticulum Quality Control
Source: PLoS One. 2010 Nov 24;5(11):e15532. doi: 10.1371/journal.pone.0015532 (PMC2991357; doi:10.1371/journal.pone.0015532)

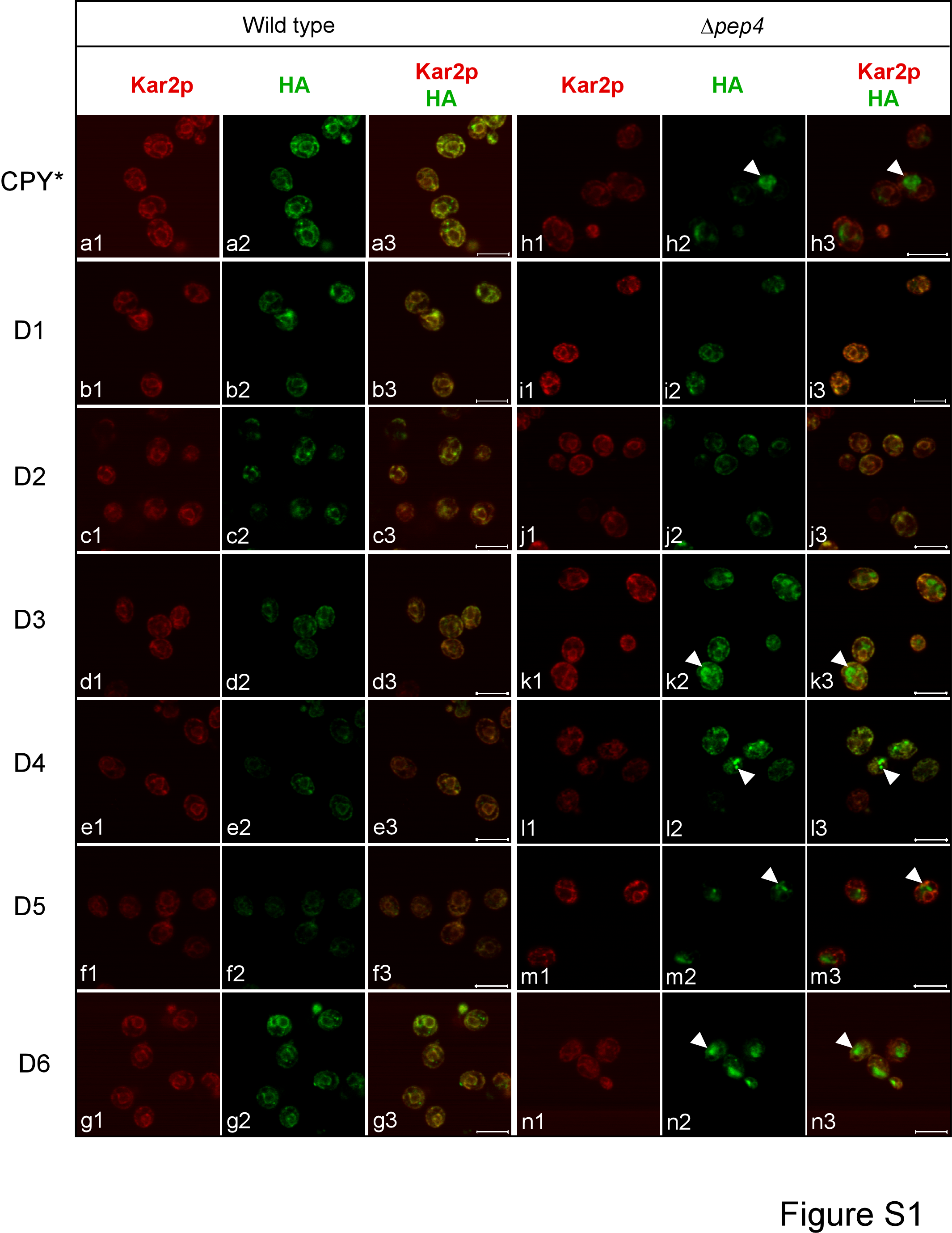

Supplement: Figure S1 — Localization of CPY* deletion variants in wild type and Δpep4 strains. Indirect immunofluorescence labeling as described in Figure 1, panels B and C. This figure shows the data set for wild type and all deletion constructs. The images shown in Figure 1 are included here to simplify viewing. Arrowheads show positions of substrate proteins localized in vacuoles. (TIF) [file pone.0015532.s001.tif]

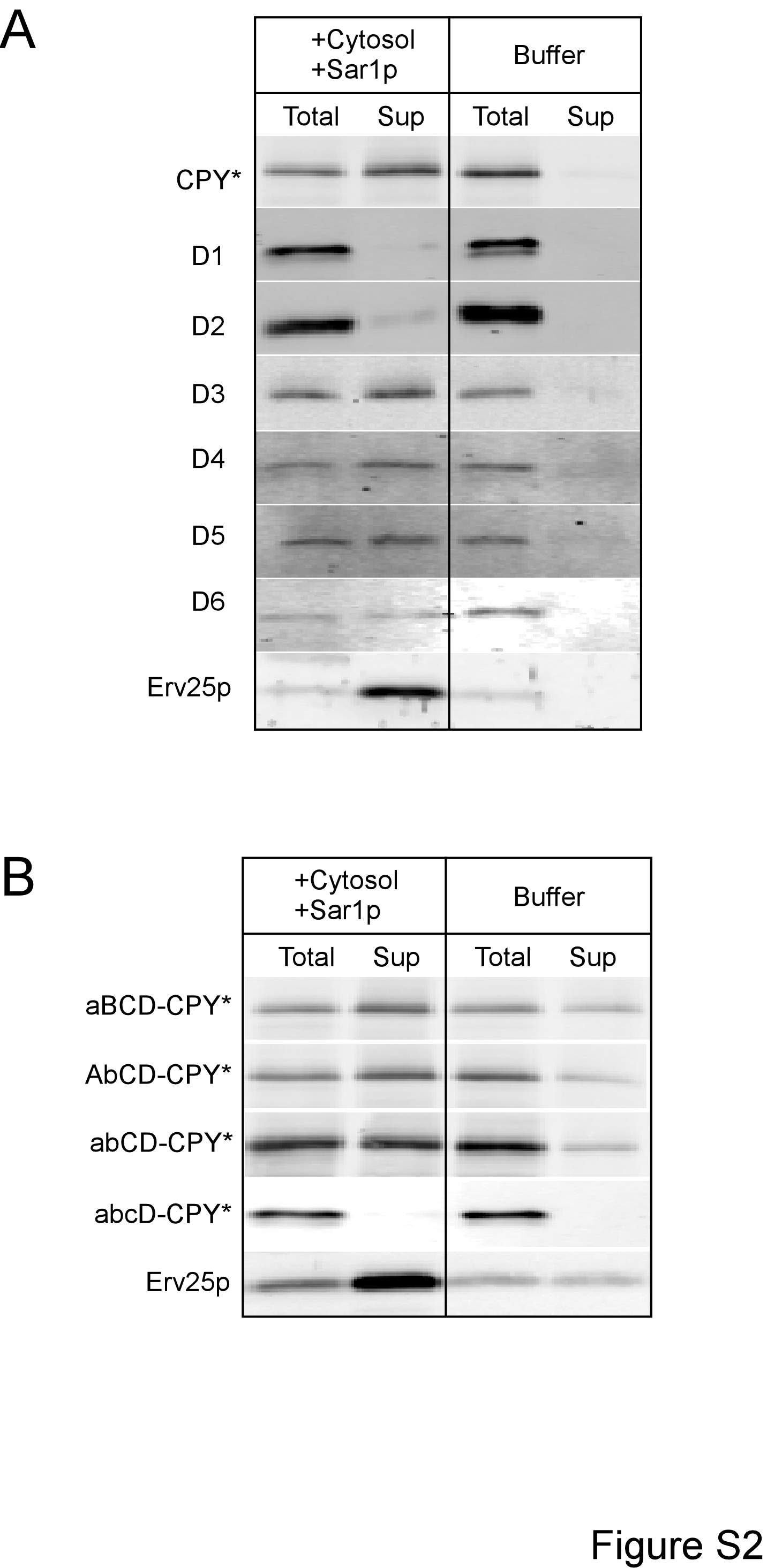

Supplement: Figure S2 — In vitro vesicle budding assays of CPY* deletion and glycan mutants. (A) Representative immunoblots of data presented in Figure 3A. (B) Representative immunoblots of data presented in Figure 5A. The COPII vesicle membrane protein Erv25p is detected as a positive control for vesicle budding in each experiment (23). (TIF) [file pone.0015532.s002.tif]

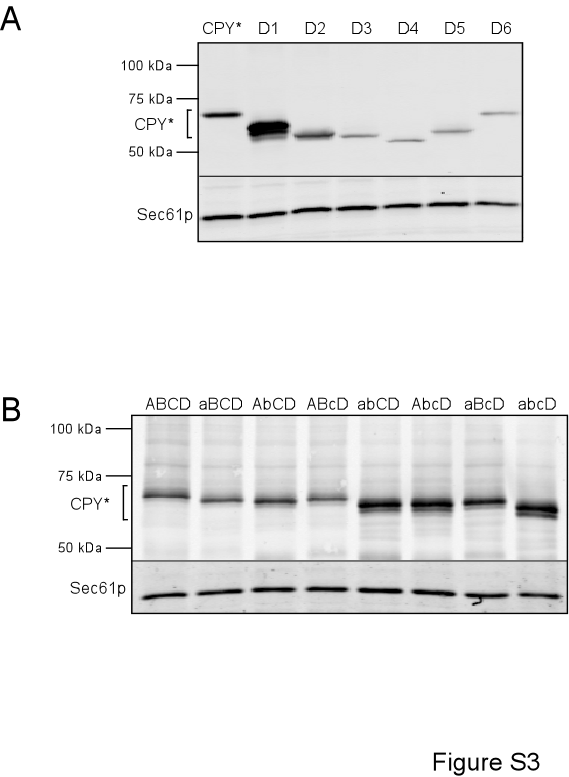

Supplement: Figure S3 — Relative expression levels of CPY* variants in wild-type cells. (A) CPY* and deletion variants were expressed in wild type cells for 6 h following galactose induction. Protein extracts from each strain were separated by SDS-PAGE and transferred onto a nitrocellulose membrane. Substrate proteins were detected using anti-HA antibody. Visualization was performed using fluorescent secondary antibodies as described in Materials and Methods . The detection of Sec61p on the same membrane was used as a loading control. The positions of molecular weight markers are indicated. (B) CPY* and glycan variants were analyzed as described in panel A. (TIF) [file pone.0015532.s003.tif]

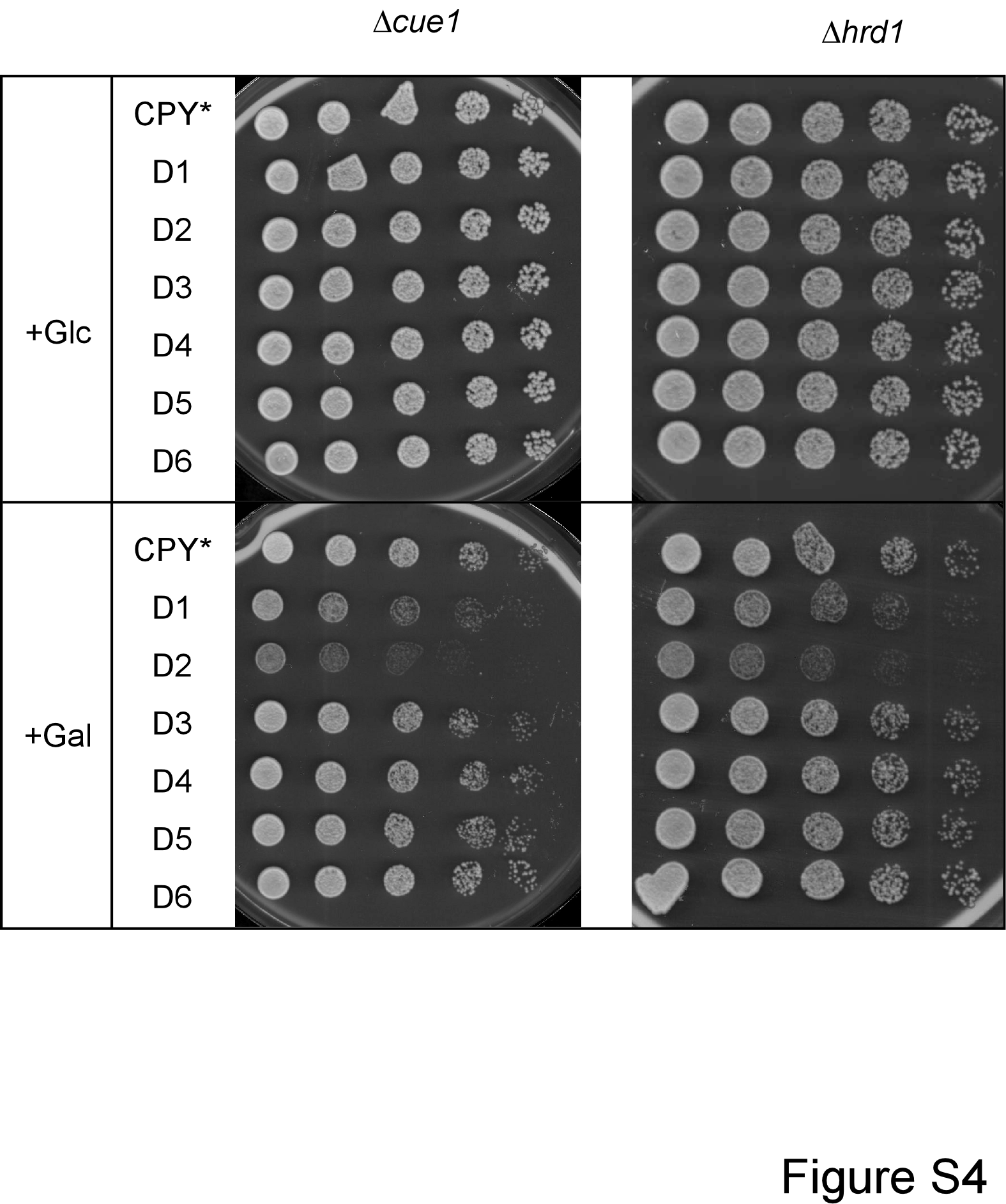

Supplement: Figure S4 — Substrate toxicity in ERAD mutants. Δcue1 and Δhrd1 cells carrying CPY* and deletion variant genes were grown overnight in culture medium containing 3% raffinose. Each culture was spotted onto agar plates as serial dilutions as described in Figure 3C. (TIF) [file pone.0015532.s004.tif]
